# Effectiveness of Robotic Systems with Dynamic Body Weight Support in Post-Traumatic Lower Limb Rehabilitation: A Systematic Review

**DOI:** 10.3390/medicina62030498

**Published:** 2026-03-06

**Authors:** Oana-Georgiana Cernea, Diana-Maria Stanciu, Roxana Pipernea, Laszlo Irsay, Viorela-Mihaela Ciortea, Mihaela Stanciu, Carmen Daniela Domnariu, Alina Liliana Pintea, Cosmina Diaconu, Florina-Ligia Popa

**Affiliations:** 1 Doctoral School, Faculty of Medicine, “Lucian Blaga” University of Sibiu, 550169 Sibiu, Romania; oanageorgiana.spatariu@ulbsibiu.ro (O.-G.C.); diana.rusu@ulbsibiu.ro (D.-M.S.); 2 Doctoral School of “Carol Davila” University of Medicine and Pharmacy, 050474 Bucharest, Romania; roxana.scheau@drd.umfcd.ro; 3 Department of Rehabilitation Medicine, “Iuliu Hatieganu” University of Medicine and Pharmacy, 400012 Cluj-Napoca, Romania; laszlo.irsay@umfcluj.ro (L.I.); viorela.ciortea@umfcluj.ro (V.-M.C.); 4 Department of Endocrinology, Faculty of Medicine, “Lucian Blaga” University of Sibiu, 550169 Sibiu, Romania; mihaela.stanciu@ulbsibiu.ro; 5 Faculty of Medicine, “Lucian Blaga” University of Sibiu, 550169 Sibiu, Romania; carmen.domnariu@ulbsibiu.ro; 6 Department of Physical Medicine and Rehabilitation, Faculty of Medicine, “Lucian Blaga” University of Sibiu, 550245 Sibiu, Romania; alina-liliana.pintea@ulbsibiu.ro (A.L.P.); cosmina.diaconu@ulbsibiu.ro (C.D.)

**Keywords:** post-traumatic pathologies, lower limb, dynamic body weight support, gait training, robotic rehabilitation

## Abstract

*Background and Objectives*: Post-traumatic lower limb injuries are frequently associated with gait impairment, reduced functional independence, and delayed recovery due to weight-bearing restrictions. Dynamic body weight support (DBWS) refers to rehabilitation technologies that provide real-time, adaptive unloading of body weight during functional tasks such as walking, enabling safer and more effective gait training. Although these robotic systems have been extensively investigated in neurological pathologies, there is a lack of evidence regarding their use in post-traumatic lower limb injuries. Therefore, this systematic review aimed to evaluate the clinical effectiveness of robotic systems incorporating DBWS in the rehabilitation of post-traumatic lower limb pathologies. *Materials and Methods*: This systematic review was conducted in accordance with the Preferred Reporting Items for Systematic Reviews and Meta-Analyses (PRISMA) guidelines, and the protocol was registered in PROSPERO (CRD420261294273). Seven major databases (PubMed, Scopus, ScienceDirect, Cochrane, Web of Science, Springer, and Wiley) were searched from inception to October 2025. Studies that were considered included patients with recent post-traumatic pathologies in the lower limbs. The methodological quality and risk of bias of the included studies were evaluated using the PEDro scale and the RoB 2 tool. *Results*: Seven studies involving 265 participants with recent post-traumatic lower limb injuries were included. The rehabilitation systems reviewed enabled early, intensive gait and balance training by reducing lower limb loading and facilitating safe performance of functional walking tasks. However, substantial heterogeneity in intervention protocols and outcome measures limited direct comparisons across studies. *Conclusions*: The findings of this systematic review suggest that DBWS interventions may enhance gait and balance recovery in individuals with post-traumatic lower limb injuries. Despite the small number of participants included, the available evidence indicates that these technologies can facilitate functional improvements during the early stages of rehabilitation and may represent a valuable adjunct to conventional therapeutic approaches. Nevertheless, further well-designed studies with larger sample sizes, standardized intervention protocols, and long-term follow-up are required to establish optimal clinical implementation strategies and to confirm the durability of treatment effects.

## 1. Introduction

Post-traumatic lower limb pathologies encompass locomotor impairments that may occur after minor trauma or major accidents and can lead to temporary or permanent functional limitations [1]. Statistics from 2019 showed that lower limb fractures are among the most common, with an incidence of 420 cases per 100,000 inhabitants [2,3]. Epidemiological data have indicated an increase in the number of accidents of various etiologies (traffic, sports, occupational or assault accidents) [4]. Moreover, Wang et al. [5] demonstrated using specialized prediction models (the best being Arima) that the number will be higher in the coming decades.

Consequently, the high incidence of lower limb fractures and severe joint injuries has led to a substantial increase in the use of total hip and knee arthroplasty, not only in post-traumatic cases but also in degenerative and secondary osteoarthritic conditions, making these procedures a major component of contemporary orthopedic and rehabilitation practice [6,7,8].

In lower limb trauma, key functions such as stability, mobility, and gait are often impaired, with repercussions on daily activities and participation in family, social, and professional life. Environmental and personal factors may also influence disability and recovery and should be considered. Pain relief and restoration of function are the primary goals in rehabilitation for post-traumatic lower limb injuries. Given the complexity of these impairments, effective management in these cases requires a multidisciplinary approach to ensure optimal recovery and minimize long-term disability of the patient [9,10].

For the rehabilitation of post-traumatic pathologies of the lower limbs, various assessment scales are initially used to establish the severity of the motor deficit. Clinical assessment involves the use of the Range of Motion (ROM) Scale [11] to assess joint mobility, the Medical Research Council (MRC) Scale [12] to assess muscle strength, the Functional Ambulation Categories (FAC) [13] to quantify functional ambulation, the Functional Independence Measure (FIM) [14] to identify the level of functional independence, the Tinetti Test (TT) [15] to appreciate balance, and assessment scales specific to daily activities, such as Activities of Daily Living (ADL) [16] and Instrumental Activities of Daily Living (IADL) [17]. The results of these functional and instrumental scales allow a comprehensive assessment of functional deficits and rehabilitation outcomes in patients with post-traumatic pathologies of the lower limbs.

In the last decade, the emphasis has been on the use of advanced technologies in rehabilitation, which play an important role by offering innovative solutions that improve recovery outcomes and treatment efficiency. These technologies offer several advantages: they may improve training intensity and safety, can be implemented more easily in structured programs, and allow long-term delivery with adjustable levels of support and progression as patients regain motor skills. The new devices used are programmable electromechanical systems that can be stationary or portable, which can be adjusted according to the individual needs of the patients. The main purpose of these systems is to retrain the lost body functions as a result of neurological or traumatic causes [18,19,20].

Neuromuscular training refers to a rehabilitation approach designed to restore coordinated interaction between the nervous and musculoskeletal systems through task-specific and feedback-driven exercises [18,19]. It typically includes components such as proprioceptive stimulation, balance and postural control exercises, progressive muscle strengthening, core stabilization, and functional movement retraining. In post-traumatic lower limb rehabilitation, neuromuscular training aims to improve joint stability, optimize muscle activation patterns, and facilitate safe and efficient gait re-education. Robotic systems incorporating dynamic body weight support may provide structured environments for delivering such neuromuscular interventions under controlled loading conditions [20,21].

Several robotic gait rehabilitation systems have been investigated across different conditions. These systems are of different types, with static or DBWS, symmetrical or asymmetrical. Some systems incorporate a treadmill on which the patient performs the training, and others only use the walking surface of the medical room. The patient’s grip system may be suspended from the ceiling or may be incorporated into the device, and the patient can explore a greater distance due to this feature. Some devices have adjustable handrails that the patient can lean on during training, and other devices are not equipped with these. All types of DBWS systems have a series of advantages and disadvantages for each pathology that has been studied, and some are under research [21,22].

Recent studies on such robotic systems with DBWS have shown that their use has increased significantly in recent years, and important aspects have been highlighted regarding the rehabilitation of neurological patients after stroke, spinal cord injury or cerebral palsy. The latest research has shown that these types of systems bring significant improvements in balance, gait and functional independence [23,24,25].

In post-traumatic lower limb pathologies, however, the literature remains limited, methodologically inconsistent, and heterogeneous. Although DBWS systems have demonstrated clear benefits in neurological populations, their application in post-traumatic lower limb rehabilitation remains insufficiently explored. Available studies include small patient samples, variable training protocols, and heterogeneous technological devices, making it difficult to interpret their results regarding the effectiveness of these systems. Moreover, most researchers focus on gait parameters, while data regarding global functionality, muscle strength, independence in daily activities, or long-term outcomes are insufficient [26,27,28].

Based on these considerations, this systematic review aims to synthesize the available evidence regarding the effectiveness of robotic systems incorporating DBWS in the rehabilitation of post-traumatic lower limb pathologies.

## 2. Materials and Methods

This systematic review was conducted in accordance with the “Preferred Reporting Items for Systematic Reviews and Meta-Analyses” (PRISMA) guidelines [29].

The review protocol was prospectively registered in the PROSPERO database under the registration CRD420261294273.

### 2.1. The Search Strategy

In line with PRISMA methodology, our research included studies indexed in the following international databases: PubMed, Web of Science, Cochrane Library, Science Direct, Springer Nature Link, Wiley Online Library and Scopus. The search covered all available literature from each database’s inception through October 2025 and was limited to studies published in English. The last electronic search was conducted on 22 October 2025. Gray literature (e.g., conference abstracts, theses, preprints, and trial registries) was not included in the search strategy.

To identify eligible studies, a systematic search strategy was implemented using keywords such as “mobile robot-based gait training”, “dynamic body weight support”, “lower limb”, “post-traumatic”, “fracture”, “rehabilitation”, and “physical therapy”, combined with the operators “AND” and “OR”. The Boolean search expression used in this research was ((“robot”) OR (“mobile robot-based gait training”) OR (“robot-assisted”)) AND ((“body weight support”) OR (“dynamic body weight support”) OR (“partial weight bearing”)) AND ((“lower limb”) OR (“lower extremity”)) AND ((“post-traumatic”) OR (“fracture”)) AND ((“rehabilitation”) OR (“physiotherapy”)).

Alternative keywords were also used in addition to the main terms “body weight support” and “mobile robot-based gait training”, such as “weight-relief system”, “weight-supported gait system”, “body-weight unloading”, “robot-guided walking training”, “robot-assisted locomotor training”, “mobile robotic locomotion training”, and “robotic gait rehabilitation with mobile platforms”. None of these alternative words revealed additional records. For each database, Boolean combinations with varied terminology were used to determine the most relevant studies. The term “neuromuscular training” was not included as a primary search keyword because the objective of this review was specifically focused on robotic systems incorporating dynamic body weight support. Pilot searches including this term did not yield additional eligible studies beyond those identified through the selected Boolean strategy. The complete search strings for each database are provided in Table 1.

This systematic search strategy was formulated to ensure a comprehensive but well-focused identification of relevant literature, thus ensuring a rigorous methodological review process. The Mendeley reference management software 2.122.0 (Elsevier, Amsterdam, Netherlands, latest version accessed on https://www.mendeley.com/) was used to include all identified articles in the databases. This software helped manage the bibliographic references, making them much easier to access and identifying duplicates. The next software that facilitated this systematic review was Rayyan (Qatar Computing Research Institute, Doha, Qatar. https://www.rayyan.ai) [30] to enhance the selection process of previously identified references. It was used to allow independent screening by two reviewers and facilitate blinding during the initial review of titles and abstracts. Screening was conducted in stages, beginning with the assessment of titles and abstracts against predefined inclusion and exclusion criteria, followed by full-text evaluation as part of the systematic review process.

### 2.2. The Study Selection

Study selection was performed independently by two reviewers (O.-G.C. and D.-M.S.) using predefined inclusion and exclusion criteria. The process involved an initial screening of titles and abstracts, after which full-text articles considered relevant were evaluated in detail. Disagreements between reviewers were addressed through discussion until agreement was reached. The screening was conducted without blinding to the authors or the journals in which the studies were published.

The inclusion criteria:

- Study design: Original research studies, including randomized controlled trials, non-randomized controlled trials, cohort studies, case–control studies, cross-sectional and prospective observational studies, pilot trials, and case series.
- Participants: Adult patients (≥18 years) diagnosed with post-traumatic lower limb pathologies, including fractures, ligamentous or tendinous injuries, joint instability, or post-surgical orthopedic conditions, diagnosed based on clinical evaluation, imaging findings, and/or surgical reports.
- Interventions: Studies evaluating robot-assisted gait rehabilitation and/or body weight support technologies, including but not limited to robotic gait trainers (e.g., Lokomat), end-effector devices, dynamic or static body weight support systems, and anti-gravity treadmills, applied alone or in combination with conventional physiotherapy.
- Comparators: Conventional physiotherapy, alternative rehabilitation interventions, or no comparator (in the case of pilot studies or case series).
- Outcomes: Studies reporting at least one functional, biomechanical, or clinical outcome, such as gait parameters, functional ambulation, functional independence, muscle strength, joint mobility, balance, pain, or quality of life.
- Setting and timing: Studies conducted in clinical or rehabilitation settings, with no restriction on follow-up duration.
- Language and publication status: Studies published in English, with full-text availability.

The exclusion criteria:

- Study design: Reviews, systematic reviews, meta-analyses, editorials, letters, conference abstracts without full text, expert opinions, clinical guidelines, and study protocols without published results.
- Participants: Studies involving pediatric populations (<18 years) or populations with primary non-traumatic neurological or metabolic pathologies (e.g., stroke, spinal cord injury, Parkinson’s disease, multiple sclerosis, diabetic neuropathy).
- Interventions: Studies evaluating only conventional physiotherapy or non-assisted gait training, without robotic systems or body weight support technologies.
- Outcomes: Studies that did not report relevant functional or gait-related outcomes or provided insufficient data for analysis.
- Study type: Animal studies, in vitro experiments, technical or engineering studies without clinical outcome assessment.
- Language and accessibility: Studies published in languages other than English or without accessible full-text versions.

### 2.3. Data Extraction

Data extraction was independently conducted by two reviewers using a standardized data extraction form developed in Microsoft Excel. The extracted information included general study characteristics, participant demographics, details of the post-traumatic lower limb pathology, intervention characteristics, comparators, outcome measures, and main findings.

For each included study, data were collected on the first author, year of publication, country of origin, study design, sample size, participant characteristics, type of post-traumatic lower limb injury, type of robotic or DBWS device, training protocol parameters, outcome measures, and key results. Any discrepancies between reviewers were resolved through discussion until consensus was achieved

### 2.4. Quality Assessment

The methodological quality and risk of bias of the included studies were assessed using two predefined appraisal tools. Two reviewers independently conducted the assessments, and any disagreements were resolved through discussion or, when necessary, by consultation with a third reviewer.

The Physiotherapy Evidence Database (PEDro) scale [31] was used to evaluate the methodological quality of randomized controlled trials, with a maximum score of 10 points and scores ≥ 6 considered indicative of high methodological quality. Additionally, the Cochrane Risk of Bias 2 (RoB 2. https://methods.cochrane.org/risk-bias-2) tool [32] was applied to randomized studies to assess potential bias across five domains: the randomization process, deviations from intended interventions, missing outcome data, measurement of outcomes, and selection of the reported results. The individual and summary assessments for each tool are described in detail in Section 3.3.

### 2.5. Statistical Analysis

Due to methodological heterogeneity and variability in outcome measures across the included studies, a quantitative meta-analysis was not feasible. Consequently, the findings were synthesized using a narrative approach to systematically summarize and interpret the available evidence.

## 3. Results

**Figure 1:**
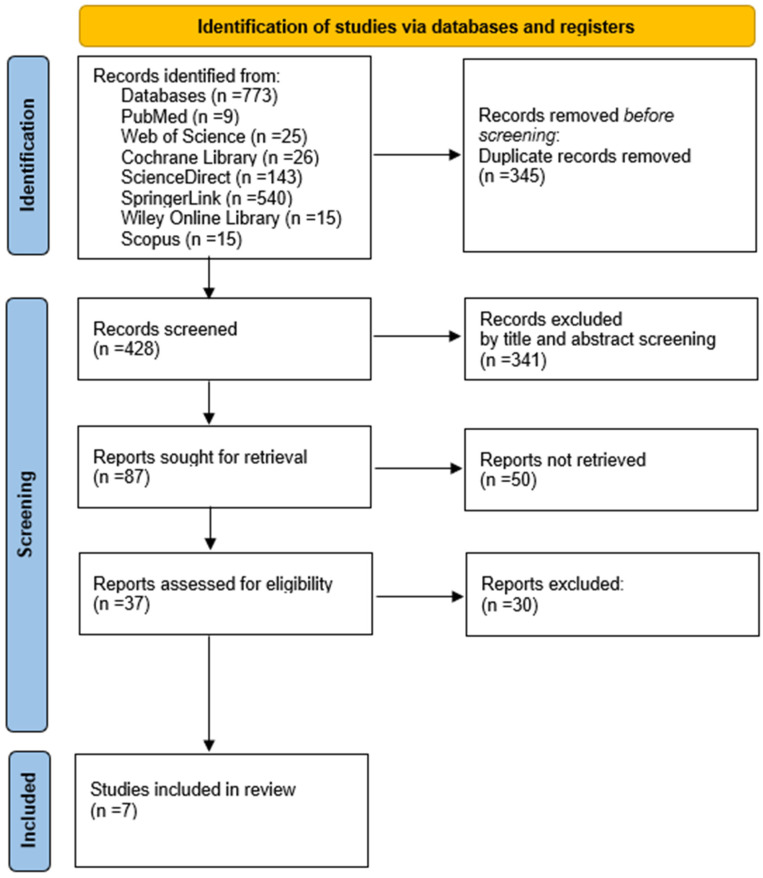
Flowchart of the study selection process according to PRISMA.

A total of 773 records were initially identified through systematic searches across multiple electronic databases (Table 1). These records constituted the basis for the subsequent screening and selection process. After the removal of 345 duplicate records, 428 unique studies remained and were screened based on titles and abstracts. During this stage, 341 records were excluded for failing to meet the predefined inclusion criteria. A total of 87 reports were subsequently sought for retrieval; however, 50 reports could not be retrieved due to the unavailability of full-text versions despite access through institutional subscriptions and interlibrary loan requests. The majority of these records were older publications or conference-related entries with unavailable full-text versions despite institutional access and interlibrary requests. The remaining 37 full-text articles were assessed for eligibility. Following full-text evaluation, 30 articles were excluded for the following reasons: inclusion of ineligible populations (e.g., non-traumatic or neurological conditions; n = 9), absence of robotic-assisted gait training or DBWS interventions (n = 7), lack of relevant gait, mobility, or functional outcome measures (n = 5), inappropriate study designs such as reviews or conference abstracts (n = 4), insufficient or incomplete data reporting (n = 3), and overlapping datasets (n = 2). Ultimately, seven studies met all eligibility criteria and were included in the qualitative synthesis. The complete selection process is illustrated in Figure 1 (Supplementary Materials PRISMA 2020 flow diagram) [29].

### 3.1. The Included Studies

After a comprehensive screening, a final selection of seven studies met the eligibility criteria and were included in this systematic review (Figure 1).

Table 2 summarizes the main characteristics of the included studies investigating DBWS systems in post-traumatic lower limb injuries.

### 3.2. The PICO Question

Eligibility for inclusion was determined by each study’s relevance to the primary research question of this systematic review: “Do robotic rehabilitation systems incorporating DBWS improve functional outcomes in individuals with post-traumatic lower limb conditions?”.

This research question was structured according to the PICO framework, as follows:

P (Population): adults with post-traumatic lower limb pathologies following surgical or conservative management;

I (Intervention): rehabilitation interventions using robotic systems incorporating DBWS;

C (Comparison): conventional rehabilitation programs, non-robotic DBWS interventions, or absence of a comparison group;

O (Outcome): improvements in gait performance, balance, functional mobility, and ambulation independence.

### 3.3. Risk of Bias

The methodological quality and risk of bias of the included studies were appraised using two validated assessment tools, selected in accordance with the respective study designs.

For randomized controlled trials (RCTs), both the Physiotherapy Evidence Database (PEDro) scale [31] and the Revised Cochrane Risk of Bias tool (RoB 2) [32] were employed.

According to the PEDro scale [31] (0–10; item 1 not included in the total score), methodological quality ranged from 4/10 to 6/10, reflecting moderate quality across trials. Two studies achieved a score of 6/10, while the remaining two scored 4/10. Lower scores were primarily attributable to the lack of blinding of participants and therapists, limited reporting of allocation concealment, and incomplete descriptions of intention-to-treat analyses, which are limitations that are common in rehabilitation trials involving device-based interventions (Table 3).

**Figure 2:**
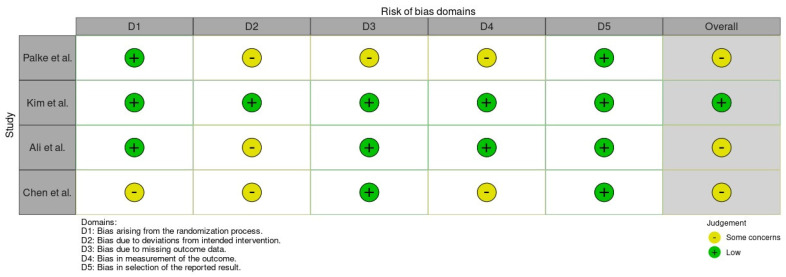
RoB 2 [32] assessment of included RCTs. Green = low risk; yellow = some concerns. While most domains showed low risk, concerns in D2 and D4 resulted in an overall judgment of some concerns for most studies.

**Figure 3:**
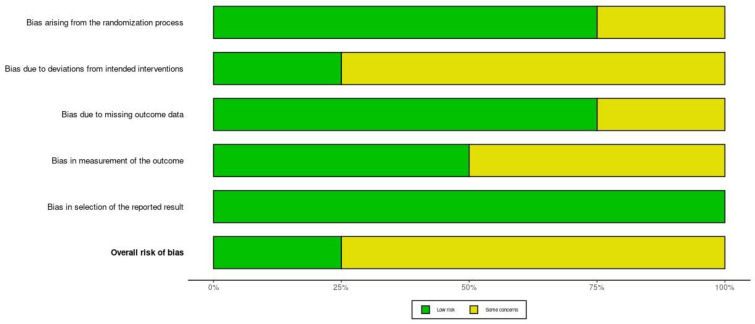
Summary of the RoB 2 [32] assessment for the included randomized controlled trials. Bars represent the proportion of studies rated as low risk (green) or some concerns (yellow) across each domain.

Using the RoB 2 tool [32], three of the four RCTs were judged to present “some concerns” regarding overall risk of bias, whereas one study was assessed as low risk. As illustrated in Figure 2 and Figure 3, concerns were most frequently identified in the domains related to deviations from intended interventions and measurement of outcomes, mainly due to the unavoidable absence of blinding and the use of partly subjective outcome measures. In contrast, the domains addressing missing outcome data and selection of the reported results were generally rated as low risk. These assessments were taken into account when interpreting the findings and drawing conclusions from the evidence.

Two reviewers independently assessed each study, and any disagreements were resolved through discussion until consensus was reached.

### 3.4. Detailed Study Description

A summary of the included studies’ main characteristics is provided in Table 4.

Palke et al. (2022, Germany) [33] conducted an open-label, prospective randomized controlled trial across three trauma centers to compare a structured anti-gravity treadmill rehabilitation program with standard rehabilitation in patients with surgically treated ankle fractures or tibial plateau fractures who were prescribed partial weight-bearing for six weeks postoperatively. Initially, 73 patients were randomized (intervention n = 37; control n = 36); at 12 months, 53 patients (intervention n = 29; control n = 24) were available for analysis. The intervention was delivered during the first six postoperative weeks, while the control group followed a standard protocol; outcomes were assessed at multiple time points from baseline/discharge through 12 months. During follow-up (12 weeks to 1 year), three participants discontinued the study (one due to travel distance and two lost to follow-up). The primary outcomes were change from baseline to 12 months in FAOS (ankle fractures) and KOOS (tibial plateau fractures); secondary outcomes included FAOS/KOOS subscores, muscle atrophy (leg circumference 20 cm above and 10 cm below the knee), range of motion, and gait performance via the Dynamic Gait Index (DGI). At one year, there were no significant between-group differences in the primary endpoints (FAOS/KOOS totals), but gait improved over time in both groups, with a significant between-group difference in DGI change from 6 weeks to 12 months favoring the anti-gravity treadmill group (*p* = 0.04); additionally, patients with tibial plateau fractures showed less muscle atrophy trends in the intervention group (e.g., thigh circumference difference persisting at 12 months, though not statistically significant at that time point). Adverse events occurred in both groups but were reported as not causally related to the intervention. Key limitations noted by the authors include a substantial dropout rate leading to small subgroup sample sizes, which reduced statistical power and widened uncertainty in subgroup findings.

Kim et al. (2020, Republic of Korea) [34] conducted a double-blinded randomized controlled trial to investigate the effects of a 4-week anti-gravity treadmill rehabilitation program compared with conventional rehabilitation in adults surgically treated for femoral fractures. A total of 34 patients were randomly allocated to an anti-gravity treadmill training group (n = 17) or a control group receiving conventional rehabilitation (n = 17). Both groups trained five times per week for 20 min over four weeks, with the intervention group performing progressive anti-gravity treadmill walking with graded BWS, while the control group followed a standardized exercise-based rehabilitation program. Primary outcomes included isokinetic muscle strength and endurance of the hip flexors and extensors, while secondary outcomes comprised surface electromyographic activity of the vastus lateralis, vastus medialis, gluteus maximus, and gluteus medius muscles, assessed before and after the intervention. Both groups demonstrated significant improvements in muscle strength, endurance, and muscle activity over time; however, between-group comparisons showed significantly greater improvements in hip extensor strength at 60°/s and significantly higher activation of the gluteus maximus and gluteus medius muscles in the anti-gravity treadmill group. No serious adverse events were reported, and no dropouts were registered. Nevertheless, the study was limited by its small sample size, heterogeneity of fracture types and injury mechanisms, short intervention duration, and the absence of longer-term follow-up, which may limit the generalizability of the findings and preclude conclusions regarding sustained functional outcomes.

Ngamwongsanguan et al. (2024, Thailand) [35] conducted a quasi-experimental pilot study at the Department of Rehabilitation Medicine, Lerdsin Hospital (Bangkok) to evaluate the effects of SensibleSTEP end-effector robotic gait training on gait and balance in older adults (≥60 years) after hip fracture surgery. Ten participants (n = 10) who had undergone hip fracture surgery (predominantly hemiarthroplasty; also, ORIF and THR) were enrolled; the cohort had a mean age of 75.6 ± 6.2 years, and all had femoral neck fractures (right/left evenly distributed). The intervention comprised 30 min sessions twice weekly for four consecutive weeks (eight sessions total), with 20 min of robotic practice per session plus preparation/rest, which was delivered with monitoring of vital signs and hip precautions; participants had also previously received conventional post-hip fracture physiotherapy from the postoperative period until hospital discharge. Information regarding participant dropout or attrition was not explicitly reported. Outcomes were assessed pre-training and after the 8th session, including FAC, Timed Up and Go (TUG), Single Leg Stance (SLS), Four Step Square Test (FSST), 10 m walk-derived gait parameters (gait speed, cadence, stride length), pain (VAS), gait aid use, and level of assistance. The study reported statistically significant improvements in FAC (*p* = 0.046), TUG (*p* = 0.005), FSS (*p* = 0.046), gait speed (*p* = 0.008), and stride length (*p* = 0.005), with non-significant positive trends for SLS, cadence, VAS, gait aid use, and assistance level. Key limitations were the small sample size, single-arm design without a control group, lack of assessor blinding, and short-term follow-up, which restrict causal inference and generalizability and support the need for a larger randomized controlled trial with longer follow-up.

Giangregorio et al. (2009, Canada) [36] conducted a pilot non-randomized controlled feasibility study in an inpatient rehabilitation setting to evaluate the feasibility and safety of body weight-supported treadmill training (BWSTT) with suspension (Pneumex Pneu-weight) as a gait retraining strategy in patients with hip fractures. A total of 21 patients with a stable hip fracture and at least 50% weight-bearing capacity were allocated to either a BWSTT group (n = 14) or a control group receiving standard physiotherapy (n = 7), with 12 participants completing the intervention and discharge assessments in the BWSTT group. Participants in the intervention group replaced usual hallway walking with BWSTT sessions lasting up to 20 min, delivered on most weekdays during inpatient rehabilitation, with an average compliance of approximately three sessions per week and individualized levels of BWS (range 0–25 kg). Primary outcomes focused on feasibility parameters, including recruitment, retention, compliance, and adverse events, while secondary outcomes assessed functional mobility and gait-related performance using the TUG, 2-Minute Walk Test (2MWT), Lower Extremity Functional Scale (LEFS), and Falls Self-Efficacy Scale (FES-I), measured at baseline, discharge, and 3-month follow-up. The study demonstrated that recruitment and implementation of BWSTT were feasible, with no serious adverse events reported; however, no significant between-group differences were observed for secondary functional outcomes. Key limitations included the non-randomized allocation, small sample size, incomplete blinding of outcome assessors, variable compliance, and a notable loss to follow-up, which limit causal inference and the generalizability of the findings and underscore the need for adequately powered randomized controlled trials.

Chen et al. (2024, China) [37] conducted a prospective randomized controlled trial to investigate the effects of standing bed training combined with early anti-gravity running table rehabilitation compared with conventional postoperative rehabilitation in patients with pronation–external rotation (PER) type III–IV ankle fractures treated surgically. A total of 52 patients (mean age approximately 40 years) were randomly allocated to an observation group (n = 26) or a control group (n = 26), with comparable baseline characteristics. Both groups received routine postoperative care, while the control group followed a staged conventional rehabilitation protocol over 8 weeks; the observation group additionally performed standing bed weight-bearing training starting on postoperative day 7 and anti-gravity running table training starting on postoperative day 28, with progressive increases in weight-bearing and walking speed, delivered once daily, five times per week. Primary outcomes included fracture healing indicators (bone scab quality score and bone mineral density), while secondary outcomes comprised ankle function (AOFAS—American Orthopaedic Foot and Ankle Society—ankle–hindfoot score), pain (VAS), ankle dorsiflexion and plantarflexion range of motion, and ankle swelling, assessed at baseline, 4 weeks, and 8 weeks postoperatively. After 8 weeks, the observation group demonstrated significantly greater improvements in bone scab quality, bone mineral density, AOFAS scores, pain reduction, and ankle mobility compared with the control group, while no significant between-group differences were observed for ankle swelling; no fixation failures or serious adverse events were reported. Dropout or attrition data were not explicitly reported. However, the study was limited by its single-center design, moderate sample size, short-term follow-up, and inclusion of only PER-type ankle fractures, which may limit the generalizability of the findings to other ankle fracture patterns.

Bodine et al. (2025, USA) [38] reported a case report describing the use of DBWS during inpatient rehabilitation in a 73-year-old woman with severe multiple trauma following a motor vehicle accident, including multiple fractures of the upper and lower extremities, ribs, pelvis, and sacrum, associated with complex weight-bearing restrictions and significant medical complications. After a prolonged acute hospitalization (22 days) complicated by respiratory failure requiring tracheostomy, the patient was admitted to inpatient rehabilitation with dependent mobility, requiring assistance from two to three therapists for transfers and ambulation. Due to her inability to safely mobilize using conventional assistive devices while maintaining upper- and lower-limb weight-bearing precautions, the patient underwent three rehabilitation sessions using a ceiling-mounted DBWS system (Vector Gait and Safety System, Bioness), initiated 26 days post-injury, which enabled sit-to-stand training, transfer practice, and overground ambulation with dynamically regulated body-weight offloading (approximately 21–33% unloading). Functional outcomes were descriptively assessed across sessions and during the inpatient stay, demonstrating progressive improvements in standing tolerance, transfer ability, walking distance (up to 25 feet with contact-guard assistance), and reduction in staff assistance required, without any adverse events or violations of weight-bearing precautions. However, the report is limited by its single-patient design, absence of standardized outcome measures, short duration of DBWS exposure, and lack of a comparator or follow-up beyond discharge, which preclude causal inference and limit generalizability; nonetheless, it provides preliminary clinical insight into the feasibility and safety of DBWS-assisted rehabilitation in patients with complex polytrauma. Although this was a single-case report without a comparator, it was included in accordance with the predefined inclusion criteria, which allowed pilot studies and case reports to provide exploratory clinical insight.

Ali et al. (2025, China) [39] conducted a prospective randomized controlled trial at a single tertiary trauma center to evaluate the effectiveness of integrating robotic-assisted mechanotherapy using the Hocoma Lokomat with conventional rehabilitation in adults with severe unstable pelvic Tile C fractures resulting from polytrauma. A total of 74 patients aged 21–65 years were randomized to either a mechanotherapy group (n = 34), which received Lokomat-assisted treadmill training combined with conventional physiotherapy, or a control group (n = 40) undergoing conventional rehabilitation alone. The robotic-assisted intervention consisted of 30–60 min sessions, three times per week for 12 weeks, with individualized gait speed and assistance levels, while both groups received matched conventional therapy in terms of duration and frequency. All 74 participants completed the 6-month follow-up, with no reported withdrawals or missing data.

Primary outcomes included pelvic functional recovery assessed using the Majeed Pelvic Score (MPS) and pain intensity measured by the VAS, while secondary outcomes comprised objective lower-limb muscle strength evaluated with the L FORCE system integrated into the Lokomat. Outcomes were assessed at baseline and at 3, 6, and 12 months postoperatively by blinded assessors. The mechanotherapy group demonstrated significantly greater improvements in pelvic function at 6 and 12 months, superior gains in walking ability and sitting tolerance, and better pain control at 6 months compared with the control group, alongside significantly higher increases in isometric hip and knee muscle strength. No participants withdrew, and no intervention-related adverse events were reported. However, the study was limited by the lack of blinding of participants and therapists, its single-center design, the moderate sample size, and potential residual confounding related to injury heterogeneity and age-related recovery differences, which may limit the generalizability of the findings.

## 4. Discussion

This systematic review synthesized evidence from seven eligible studies investigating robotic-assisted and DBWS-based gait rehabilitation systems in patients with post-traumatic lower limb and pelvic injuries. Overall, the included studies suggest that interventions incorporating DBWS interventions may improve gait performance, functional mobility, and muscle strength; however, these findings must be interpreted cautiously due to methodological limitations and clinical heterogeneity across studies.

Substantial variability was observed in study design, sample size, intervention protocols, and outcome measures, limiting comparability and precluding quantitative synthesis. Therefore, the following discussion critically evaluates the findings of the included studies in relation to their methodological quality, reported outcomes, and clinical applicability. For contextual purposes, selected external evidence is referenced to situate the findings within the broader rehabilitation literature.

For clarity, the discussion is structured to address the characteristics of post-traumatic lower limb and pelvic pathologies, followed by an evaluation of the BWS-based and robotic rehabilitation interventions and their effects on gait and functional outcomes. Finally, the methodological limitations of the included studies are discussed, and directions for future research are proposed to support the development of standardized and evidence-based rehabilitation protocols for post-traumatic lower limb injuries.

### 4.1. Pathological Characteristics of Post-Traumatic Lower Limb Conditions

The pathological characteristics of the studies included in this systematic review reflect a wide spectrum of post-traumatic lower limb injuries, ranging from isolated fractures to complex pelvic fractures and multiple trauma. These types of injuries are frequently associated with significant morbidity, prolonged disability, and high risk of recurrent injury. Consistent with broader epidemiological evidence, older adults with hip fractures face particularly poor outcomes: one-year mortality is around 25%, and nearly half of survivors experience long-term loss of independence or require assistive devices for mobility [40].

Several studies focused on isolated lower limb fractures, particularly femoral, hip, and ankle fractures. Patients with femoral fractures commonly exhibited reduced muscle strength, impaired neuromuscular activation, and limitations in early ambulation following surgical fixation, as reported in randomized and controlled trials evaluating anti-gravity treadmill training [33,34]. Similarly, the hip fracture population, predominantly older adults, presented with marked gait instability, balance deficits, and increased functional dependence, underscoring the need for safe and progressive gait retraining during the early postoperative phase [35,36]. In ankle fracture patients, localized impairments such as pain, joint stiffness, gait asymmetry, and delayed restoration of full weight bearing were frequently observed, particularly during early rehabilitation stages [37].

More severe traumatic profiles were addressed in studies involving unstable pelvic Tile C fractures and multiple trauma patients. These conditions were characterized by extensive skeletal and soft tissue injuries, prolonged restrictions on weight bearing, and pronounced deficits in mobility and postural control, necessitating carefully individualized rehabilitation strategies [38,39]. In such cases, DBWS systems enabled early neuromuscular activation and assisted ambulation while minimizing mechanical stress on healing structures.

Across all pathological categories, a common clinical challenge was balancing early mobilization with protection of injured tissues. Pain, fear of loading, and biomechanical constraints related to fracture stability and surgical fixation often limited conventional rehabilitation approaches. In this context, BWS and robotic-assisted gait systems were employed to facilitate early gait training under controlled loading conditions, enhance patient confidence, and promote functional recovery [33,34,36,37,38]. Although injury location and severity varied, the consistent presence of gait impairment and limited weight-bearing tolerance across studies supports the applicability of BWS rehabilitation in post-traumatic lower limb pathologies.

### 4.2. Instruments and Rehabilitation Systems Used

A variety of robotic and mechanically assisted systems with DBWS were employed across the studies to facilitate gait training and rehabilitation. Although all interventions aimed to promote early mobilization and functional recovery after post-traumatic lower limb injuries, substantial differences were observed in the type of systems used, their technical characteristics, and their mode of application. Studies not meeting the eligibility criteria are cited only to contextualize the findings where direct evidence from the included studies is limited.

Anti-gravity treadmill systems, most frequently represented by the AlterG^®^ treadmill, were the predominant devices used across studies [33,34,36,37]. These devices enclose the lower body in an airtight chamber and use positive air pressure to offload a precise percentage of body weight during walking. By reducing effective gravity on the limbs, an anti-gravity treadmill allows patients to ambulate with less joint load and pain, while maintaining a normal gait pattern. For instance, the AlterG^®^ treadmill used by Kim et al. [34] provided up to 75% weight support initially, automatically adjusting chamber pressure as the patient’s legs moved to ensure consistent unloading. This enabled patients with femoral fractures to perform gait training well before full weight-bearing was medically permissible.

In complex cases such as multiple trauma, DBWS systems (e.g., Bioness Vector) enable adaptive unloading during gait training through an overhead harness that supports a programmed percentage of body weight and provides fall protection. These ceiling-mounted systems dynamically adjust support in real time, facilitating safe sit-to-stand transitions and ambulation despite balance instability and fluctuating weight-bearing tolerance. As reported by Bodine et al., use of a Vector system allowed a 73-year-old polytrauma patient with pelvic and extremity fractures to safely practice transfers and short-distance walking with a single therapist, substantially reducing staff requirements and fall risk compared with conventional rehabilitation approaches [38].

Robotic gait orthoses have also been incorporated into trauma rehabilitation, most notably the Hocoma Lokomat, an exoskeleton-based treadmill system providing adjustable guidance force and partial BWS. In patients with unstable pelvic fractures, the Lokomat enabled early and safe mobilization by guiding the lower limbs through a physiological gait pattern while preventing collapse and maintaining alignment, with programmable control of gait speed, step length, and joint range of motion. Its integrated force sensors and performance-monitoring software (L-Force) further allowed objective tracking of patient progress [39].

Another category is end-effector gait trainers, such as the SensibleSTEP device used by Ngamwongsanguan et al. [35]. Instead of an exoskeleton, this end-effector robot has two footplates that move in an elliptical trajectory, simulating stepping while the patient’s feet are strapped on. SensibleSTEP incorporates adjustable vertical and horizontal BWS, a supported harness system, and allows for tuning of gait speed and step length. In practice, this device enabled frail elderly hip-fracture patients to practice walking with partial weight support and feedback on proper gait mechanics.

Several studies combined novel devices with conventional aids. Chen et al. [37] used a tilt-table device beginning one week after ankle surgery to introduce partial weight-bearing in a static upright posture. By postoperative week 4, patients progressed to anti-gravity “running table” training (an AlterG^®^ treadmill) for dynamic gait exercise. This staged approach (from supported standing to supported walking) exemplifies how multiple instruments can be used sequentially to safely accelerate weight-bearing.

Beyond the studies included in this review, these devices have demonstrated their benefits in multiple studies published in recent years, supporting their importance in gait rehabilitation across a wide range of pathologies. One study reported improvements in walking capacity and changes in the corticospinal tract reflecting neuroplasticity following AlterG^®^ training in children with cerebral palsy [41].

Moreover, Huber et al. [42] observed significant benefits in both motor and autonomic functions after DBWS training, along with a significant improvement in balance parameters, thereby reducing fall risk and supporting reintegration into activities of daily living [43]. Similarly, external evidence has highlighted the clinical relevance of customized BWS systems with a developed platform integrated that supports the role of BWS technologies in facilitating early mobilization, reducing joint loading, and improving functional recovery in vulnerable post-traumatic populations [28].

In summary, the rehabilitation systems across studies share a common goal: provide dynamic unloading of the lower limbs to permit early, intensive practice of gait and balance. Whether via an air-pressure treadmill, an overhead robotic harness, or a foot-plate gait trainer, these technologies create a protected environment in which patients can perform repetition-rich ambulatory tasks that would be impossible or unsafe under full gravity so soon after injury.

### 4.3. Intervention Protocols and Training Parameters

The intervention protocols applied across the included studies varied considerably in terms of training duration, session frequency, progression strategies, and timing of rehabilitation initiation. Despite these differences, all protocols were designed to facilitate early gait re-education while ensuring adequate protection of healing tissues following post-traumatic lower limb injuries.

In most studies, BWS training was initiated as early as medically permissible after injury or surgery. Palke et al. [33] introduced anti-gravity treadmill training within the first six postoperative weeks in patients with tibial plateau and ankle fractures, replacing conventional walking exercises with progressively increased weight-bearing ambulation. Similarly, Kim et al. [34] implemented a structured 4-week anti-gravity treadmill protocol in hip fracture patients, consisting of 20 min sessions performed five times per week, with a systematic reduction in BWS from >75% in Week 1 to near-full loading by Week 4. This stepwise progression enabled safe and gradual reloading of the injured limb, ensured adherence to surgical weight-bearing restrictions, and facilitated confident ambulation at full body weight by the end of the intervention.

Low-impact sessions of 20–30 min were commonly used to balance intensity with patient fatigue and safety. Ngamwongsanguan et al. [35] showed that twice-weekly 30 min robotic gait training over four weeks produced functional gains despite modest frequency. In inpatient settings, Giangregorio et al. [36] reported about three treadmill sessions per week, while Bodine [38] used short, frequent DBWS-assisted overground walking once upright tolerance was achieved. Ali et al. [39] applied Lokomat training three times weekly for 30–60 min alongside standard therapy. Chen et al. [37] progressed patients from tilt-table standing (postoperative day 7) to AlterG^®^ treadmill training at week 4, with individualized load progression. Importantly, none of these early weight-bearing approaches negatively affected fracture healing, and improved bone consolidation was reported in the intervention group.

All in all, the optimal training parameters are still being refined, but the successful programs to date share a philosophy of early start, gradual progression, regular frequency, and integration with standard rehab. Preliminary external evidence even suggests that higher training dosages (e.g., daily sessions with substantial BWS in the first 4–6 weeks) may confer advantages in muscle strength and gait recovery, a point that merits further investigation in larger trials.

### 4.4. Objective Posturographic and Instrumented Gait Assessments

A key advantage of robotic and instrumented rehabilitation systems is their ability to provide objective, quantitative measures of gait and balance. Ngamwongsanguan et al. [35] assessed spatiotemporal gait parameters using a 10 m walk test on a sensorized mat and reported post-training improvements in gait speed and stride length after eight robotic sessions, indicating a more normalized gait pattern, with a positive trend toward increased cadence. Objective balance outcomes were also reported: in the SensibleSTEP pilot, performance on the FSST improved significantly, reflecting better dynamic balance and agility, while SLS time increased from 4.1 s to 7.5 s in the robot-trained group, suggesting clinically meaningful gains in static balance despite the lack of statistical significance.

Instrumented gait analysis provided detailed insight into neuromuscular adaptations in studies using integrated robotic systems. Ali et al. [39] used Lokomat L-FORCE assessments to quantify isometric hip and knee strength, reporting greater muscle force gains in the robotic training group. Kim et al. [34] combined isokinetic dynamometry and surface EMG (electromyography) to evaluate recovery after four weeks of intervention, showing significantly larger improvements in the anti-gravity treadmill group, including higher peak hip extensor torque (d = 0.78, *p* = 0.029) and markedly increased gluteus maximus and medius activation (*p* < 0.001). These objective findings were attributed to early, safe practice of single-limb support under partial weight-bearing and support the conclusion that DBWS training produces measurable improvements in muscle output and gait mechanics.

Posturographic measures were rarely reported in the reviewed studies, beyond standard clinical balance tests, but emerging research is beginning to address this gap. Although not included in the present review, a large Italian RCT involving 195 patients is incorporating the TYMO^®^ force platform to quantify postural stability and an instrumented treadmill (Walker View^®^ with load cells and 3D motion capture) to longitudinally assess gait kinematics, reflecting a broader shift toward instrumented gait analysis as an outcome measure [44]. These systems allow both real-time patient feedback (e.g., step symmetry and weight distribution) and precise, objective monitoring of gait recovery. This emphasis on objective assessment observed in the included studies is consistent with recent recommendations by Xia et al. (2025), who advocate integrating 3D motion analysis or wearable sensors in future studies to better substantiate functional improvements [45].

While traditional clinical scales still dominate outcome reporting, the use of DBWS technology opens the door for richer biomechanical assessment. Early results show improvements in quantifiable domains such as gait speed, balance time, muscle strength, and activation patterns, which underpin the functional gains seen in patients.

### 4.5. Clinical Balance and Gait Outcome Measures

All seven studies reported conventional clinical outcomes related to gait and balance, demonstrating greater functional improvements with DBWS-based robotic training compared with standard care, though with variability across measures and sample sizes. In the SensibleSTEP pilot, FAC scores improved significantly after four weeks, with participants progressing from assisted walking (FAC 3) to supervised or independent ambulation, and 7 of 10 achieving full community walking (FAC 5; median FAC 4→5, *p* = 0.046). Timed functional performance showed parallel gains: TUG scores improved from 31.3 s to 19.7 s (*p* = 0.005), a clinically meaningful reduction associated with lower fall risk and improved balance, mobility, and gait confidence, consistent with findings from other robotic gait training studies.

Balance-specific outcomes also favored DBWS-based training. In the SensibleSTEP study, FSST performance improved significantly, with participants progressing from inability or times of >15 s to successful completion within ≤15 s (*p* = 0.007), indicating enhanced dynamic balance and directional control. Although Giangregorio et al. [36] found no significant between-group differences on the FSE-I at discharge, this feasibility study was underpowered and focused primarily on safety. Qualitative reports nonetheless suggested reduced fear during gait training with harness support, supporting the notion that improved balance performance may be accompanied by greater confidence in ambulation.

Limb-specific clinical gait outcomes also supported DBWS-based interventions. Chen et al. [37] reported significantly higher AOFAS Ankle–Hindfoot scores after eight weeks in patients with PER ankle fractures who received early standing and anti-gravity treadmill training, compared with standard rehabilitation (mean difference ~9–10 points). These higher scores, reflecting reduced pain and improved function, were accompanied by superior recovery of ankle dorsiflexion, a key determinant of normal gait. The authors attributed these gains to early partial weight-bearing on the anti-gravity treadmill, which likely limited joint stiffness and accelerated restoration of ankle motion.

For proximal injuries, multidimensional outcome scales highlighted the benefits of robotic and DBWS-based rehabilitation. Ali et al. [39] used the MPS and reported consistently higher scores in the Lokomat group throughout rehabilitation (≈91 vs. ≈88 in controls), along with lower pain levels at 6-month follow-up, reflecting clinically meaningful functional gains. In Palke et al.’s [33] anti-gravity treadmill study, overall PROMs (FAOS, KOOS) did not differ when all patients were pooled, but stratified analysis showed significant benefits in patients with more severe injuries. Specifically, those with tibial plateau fractures demonstrated superior KOOS Symptoms and QOL scores at one year in the anti-gravity group, exceeding minimal detectable change thresholds. The same study also found greater improvement in DGI scores between 3 and 12 months postoperatively in the anti-gravity group (median +3 vs. +1; *p* = 0.04), indicating better advanced mobility and reduced fall risk. Collectively, these findings support the conclusion that robotic and DBWS interventions provide superior improvements in functional mobility and patient-reported outcomes compared with conventional rehabilitation, particularly in patients with higher initial impairment.

Importantly, none of the included studies reported deterioration in clinical outcomes or safety concerns related to robotic or DBWS interventions. The absence of adverse events or falls during training likely enabled greater patient engagement and facilitated functional recovery. In Bodine’s case study [38], DBWS-supported therapy led to marked improvements on the Functional Independence Measure, with the patient progressing from total dependence to partial or supervised assistance in bed mobility, transfers, and short-distance ambulation within weeks. These gains highlight the clinical relevance of DBWS training, as even modest improvements in standing and walking ability can represent a substantial restoration of independence in severely impaired patients.

Overall, the compiled evidence strongly supports the effectiveness of DBWS and robotic systems in improving gait and balance outcomes after lower-limb trauma, beyond what standard rehabilitation alone typically achieves.

### 4.6. Risk of Fall and Safety Outcomes

Fall prevention and safety are central goals of rehabilitation, and robotic DBWS systems appear to address both by providing a secure training environment while improving balance. Across studies, no serious adverse events were reported. Giangregorio et al. [36] documented no falls or near-falls during more than 100 BWSTT sessions in hip fracture patients, while Bodine’s case study [38] showed that a dynamic harness enabled safe practice of standing, transfers, and walking in a high-risk patient, with automatic balance support also reducing therapist injury risk. These safety features allowed more challenging and earlier gait activities than would otherwise be feasible, facilitating faster functional gains. Ali et al. [38] similarly reported that the Lokomat offered a safe early gait-training environment for polytrauma patients, with no meaningful device-related complications, indicating good overall tolerability of DBWS systems.

Although most studies were not designed or powered to measure post-discharge fall incidence, several assessed validated surrogate markers of fall risk. Clinically meaningful reductions in Timed Up and Go time (from ~31 s to ~20 s) indicate a shift from high to moderate fall risk in elderly patients. Similarly, improvement in the FSST to within the normal range (≤15 s) reflects enhanced agility and balance relevant to fall avoidance. Ngamwongsanguan et al. [35] also reported upward shifts in Functional Ambulation Category, with 70% of participants reaching FAC 5 (independent ambulation on all surfaces) by study end, a level associated with lower fall risk. Notably, no patients regressed or experienced falls during the intervention period, and those with prior fall history did not fall again during training, suggesting improved stability.

Fear of falling, an important psychological contributor to fall risk, was also indirectly addressed. Although pilot studies did not show significant differences in formal fear-of-fall scores, patient confidence improved alongside functional recovery. In Bodine’s case study [38], the patient progressed from being unable to attempt standing pivot transfers due to fear to walking 25 feet with supervision after several DBWS sessions, suggesting restored mobility self-efficacy within a secure training environment. Larger trials, such as the ongoing Italian RCT, are explicitly measuring fear of falling using the FES-I [46]. Although not specifically assessed in all included studies, this focus is supported by prior geriatric research demonstrating that improved balance performance is associated with reduced fear of falling and greater post-fracture participation [47].

From a clinical safety perspective, DBWS interventions did not compromise primary recovery and may have enhanced overall rehabilitation safety. Chen et al. [37] reported no increase in wound, fixation, or hardware-related complications with early partial weight-bearing, nor any rise in common postoperative medical events. Early mobilization may, in fact, reduce complication risk by limiting prolonged bed rest. Ali et al. [39] similarly observed better functional outcomes and fewer secondary complaints in the Lokomat group at follow-up, including lower pain levels and greater strength. Across studies, DBWS systems were applied under strict therapist supervision and safety protocols, which likely contributed to the absence of adverse events and the consistently favorable safety profile.

In a post-acute rehabilitation cohort, use of the Andago overground DBWS trainer was associated with a reduction in fear of falling during gait re-education sessions compared with conventional overground walking, as participants reported lower perceived fear of falling at 10 and 20 min while walking with the device [46].

DBWS systems improve rehabilitation safety and functional outcomes by enabling safe gait and balance training in high-risk patients, leading to better balance, reduced pain, increased confidence, and a likely reduction in future fall risk without compromising patient safety.

### 4.7. Impact of Functional Independence and Quality of Life

Restoring functional independence and improving quality of life (QOL) are the ultimate goals of rehabilitation. While many of the measures discussed (walking speed, FAC level, etc.) relate to specific abilities, it is vital to consider the broader impact on patients’ daily living and well-being. The current evidence, though still emerging, suggests that robotic DBWS interventions can yield meaningful improvements in these domains, especially for those with severe mobility limitations after trauma.

Several studies included patient-reported or composite functional indices that reflect independence in daily activities. For instance, the KOOS and FAOS used by Palke et al. [33] each contain subscales for ADL and sports/recreation, as well as a knee/ankle-related QOL subscale. At one-year follow-up, there were notable between-group differences in favor of anti-gravity training: among tibial plateau fracture patients, the intervention group reported better ADL function and much higher knee-related QOL (mean ~71 vs. 59, as mentioned). While the trial was not powered to detect differences in every subscore, the fact that QOL and symptom relief were better with the treadmill is clinically meaningful. Patients essentially perceived fewer knee problems in their daily life, which likely corresponds to greater confidence walking and an earlier return to the activities they value.

Similarly, Ali et al.’s [39] pelvic fracture study, by using the MPS, indirectly captured return to functional roles (the scoring of which awards points for work status and sitting ability). The Lokomat group’s higher MPS implies that more patients in that group regained the capacity to sit comfortably for long periods and possibly to resume work or homemaking tasks. Though a detailed breakdown was not provided in the summary, a mean score in the 90s usually corresponds to an “excellent” outcome, indicating minimal functional limitation in most daily tasks.

QOL after trauma is influenced by physical, emotional, and social factors. Although generic QOL instruments were not used, improvements in pain, mobility, and independence observed with DBWS training represent meaningful QOL gains. Lower pain scores are clinically important, as reduced pain supports daily function and sleep. The functional gains illustrated in Bodine’s [38] case highlight how improved mobility can determine discharge home versus institutionalization, consistent with evidence that ambulatory capacity predicts post-hip fracture living outcomes.

Although not included in this review, a prospective longitudinal observational study about hip fracture patients before and after surgery that employed specific instruments for QOL assessment, such as the SF-36 and WHOQOL-BREF, demonstrated significant improvements in functional and psychological domains three months after the fracture, highlighting the positive impact of treatment and rehabilitation on patients’ QOL [47].

In Giangregorio et al.’s study [36], even though the treadmill group’s functional outcomes did not significantly exceed those of controls by discharge, they observed that those who walked with BWS tended to have higher gait speeds and a faster improvement trajectory by 3 months. With a larger sample, it is conceivable that this would manifest as higher rates of independent living or community ambulation in the BWS group.

Psychological well-being and confidence are key components of QOL after trauma, as fear of falling and depression can markedly impair recovery. By enabling safe early ambulation and improving balance, DBWS systems help reduce kinesiophobia and restore confidence. In the SensibleSTEP trial, patients reported greater walking security, reflected by reduced reliance on walking aids and assistance. Such gains in autonomy support self-esteem and social participation [35]. Additionally, DBWS-assisted interventions may shorten rehabilitation duration, with clear QOL benefits. In another study that used the SWalker, robotic training reduced time to independent ambulation by 46 days and required substantially fewer sessions than conventional therapy, limiting prolonged dependency and disability. As concluded by Costa et al., robotic platforms effectively enhance autonomous gait while accelerating rehabilitation, thereby contributing to improved QOL [28].

In summary, the current evidence points to substantial benefits of DBWS-assisted rehab on functional independence and aspects of QOL. Patients experience less pain, greater ability to perform ADLs and ambulation, and likely more social independence as a result. These systems help patients reclaim their autonomy faster, whether measured in the ability to walk without human help, the confidence to move without fear, or the simple joy of being able to stand and take steps after being immobilized. A recent external systematic review in Heliyon (2024) concluded that anti-gravity treadmill rehabilitation in musculoskeletal conditions led to greater improvements in pain relief, gait functionality, and even fracture healing, with patients who underwent such training showing better overall outcomes than those with conventional rehab [48].

### 4.8. Limitations of the Current Evidence

Despite the encouraging findings, this body of evidence has important limitations that temper the conclusions. First, sample sizes are small in most studies, with three of the seven included papers being pilot trials (10 patients or fewer) or case reports. Small samples reduce statistical power, may overestimate effect sizes, and limit subgroup analyses.

Many studies were single-center and lacked participant blinding, as the nature of the intervention made blinding challenging. While outcomes like gait speed are objective, patient-reported measures could be influenced by placebo or expectation effects. That said, the fact that several trials found no placebo-driven differences in subjective scores (e.g., FAOS/KOOS total scores were similar between groups) provides some reassurance that bias was minimal.

Another limitation is the heterogeneity of protocols and outcome measures. As seen, interventions ranged from 4 weeks of daily treadmill sessions to 8 weeks of weekly sessions to 6+ weeks of inpatient programs. Outcome measures were diverse: one study might focus on isokinetic strength, another on a clinical score, another on gait kinematics. The review did affirm generally positive trends, but the lack of uniform metrics makes it hard to calculate pooled effect sizes.

Short follow-up duration is another common limitation. Only two studies followed patients for 6 or 12 months post-injury. Most others assessed outcomes immediately after the intervention period (4–8 weeks, or at hospital discharge). Thus, we have limited data on whether the initial advantages of robotic training persist long-term or translate into tangible differences in ultimate recovery. Without extended follow-up, these trajectories remain uncertain. Chen et al. [37] acknowledge not performing a later-stage follow-up to see if the early treadmill group maintained superior ankle function or if both groups converged after full rehabilitation. The need for longitudinal studies beyond discharge is clear and is echoed by others.

Methodologically, several studies were non-randomized or lacked a true control group. Giangregorio et al.’s [36] trial was nonrandomized (patients self-selected or were selected into BWSTT vs. control), raising the possibility of selection bias (perhaps therapists chose more motivated patients for treadmill walking). Bodine’s report was a case study without a control, which is low on the evidence hierarchy [38]. Even the RCTs often had practical limitations: Palke’s was an open-label trial, and although Kim’s and Ali’s were randomized, blinding of outcome assessors was not mentioned [33,34,39].

Another subtle limitation is the learning curve and resource requirement for implementing these technologies. Ali et al. [39] noted that all Lokomat operators underwent 20 h of training and certification. The effectiveness of the intervention could depend on staff proficiency—a factor rarely accounted for in analysis. If a study’s results are achieved at a specialized center with highly trained personnel, they might not immediately generalize to a typical clinic without similar training. High cost and limited availability of robotic devices can also be seen as an external limitation: none of the studies performed a cost-effectiveness analysis, but it is an implicit consideration for widespread adoption.

The exclusion of gray literature and restriction to English-language publications may have introduced publication bias and affected the overall scope of the retrieved evidence. Overall, although the findings are encouraging, the available studies remain constrained by small sample sizes, methodological heterogeneity, and short follow-up durations, and they predominantly cover select patient groups. Future research must address these issues to strengthen the robustness of conclusions.

### 4.9. Future Research Directions and Clinical Implications

Building on the findings and limitations above, several clear avenues for future research and important clinical implications emerge. High-quality, large-scale trials are a top priority. As recommended by Xia et al. (2025) [45], we need larger multicenter RCTs to improve the robustness and generalizability of results. Enrolling a more diverse patient population across multiple hospitals will help determine if the benefits of robotic BWS seen in single-center studies hold true broadly and will allow subgroup analyses to identify which patients benefit most. Future trials should establish consensus on training parameters—such as optimal session duration, frequency per week, and total weeks of intervention—as current studies varied widely. Kim et al. [34] noted it remains unclear “how long and how many sessions per week” are ideal to achieve full weight-bearing ability in hip fracture rehab.

Technologically, the field is evolving, so integrating new features like virtual reality (VR) or perturbation training into DBWS systems is a compelling direction. Some modern BWS treadmills can deliver lateral perturbations or simulate uneven terrain to train reactive balance (one study protocol involves a perturbation module on a ceiling BWS track) [49]. Investigating whether adding perturbation or VR-enhanced environments (for cognitive engagement) further improves outcomes would be worthwhile. A recent scoping review highlighted VR’s potential to enhance musculoskeletal rehab by increasing patient engagement and providing real-time feedback [50]. Combining VR with BWS robotics (for example, a patient in a harness walking through a virtual obstacle course) could synergistically improve both motor and cognitive aspects of balance; research in this area is just beginning [51].

Looking further ahead, protocol standardization and training for staff will be essential for broad implementation, as highlighted by Xia et al. [45].Certification programs should become more widespread so that more therapists are skilled in these technologies.

Overall, future research should focus on confirming and refining the promising results seen so far through larger trials, longer follow-ups, standardized outcome sets, and exploration of technology combinations.

## 5. Conclusions

The clinical takeaway at this stage is that robotic and DBWS systems represent a valuable advancement in orthopedic trauma rehabilitation. They allow clinicians to provide intensive, task-specific therapy in a safe manner, which appears to accelerate recovery of gait and balance. As the population ages and the incidence of fragility fractures rises, such innovative approaches may become increasingly important to improve outcomes and reduce the burden of disability. Ongoing and future studies will clarify how best to integrate these tools into routine care. Based on the current evidence, DBWS gait training shows promise as a potential adjunct to conventional rehabilitation in selected patients with severe lower limb injuries.

## Figures and Tables

**Table 1:** Number of articles retrieved from each database using the Boolean search string.

Databases	Boolean Search String	Filter Applied	Number of Results
PubMed	(“robot” OR “mobile robot-based gait training” OR “robot-assisted”) AND (“body weight support” OR “dynamic body weight support” OR “partial weight bearing”) AND (“lower limb” OR “lower extremity” OR “leg” OR “knee” OR “ankle” OR “hip”) AND (“trauma” OR “post-traumatic” OR “fracture” OR “injury”) AND (“rehabilitation” OR “physiotherapy”)	English, Humans, Adult	9 results
Web Of Science	(“robot” OR “mobile robot-based gait training” OR “robot-assisted”) AND (“body weight support” OR “dynamic body weight support” OR “partial weight bearing”) AND (“lower limb” OR “lower extremity” OR “leg” OR “knee” OR “ankle” OR “hip”) AND (“trauma” OR “post-traumatic” OR “fracture” OR “injury”) AND (“rehabilitation” OR “physiotherapy”)	Rehabilitation, English	25 results
Cochrane Library	(“robot” OR “mobile robot-based gait training” OR “robot-assisted”) AND (“body weight support” OR “dynamic body weight support” OR “partial weight bearing”) AND (“lower limb” OR “lower extremity” OR “leg” OR “knee” OR “ankle” OR “hip”) AND (“trauma” OR “post-traumatic” OR “fracture” OR “injury”) AND (“rehabilitation” OR “physiotherapy”)	Trials	26 results
Science Direct	(mobile robot-based gait training OR “robot-assisted”) AND (“body weight support” OR “dynamic body weight support”) AND (“lower limb” OR “lower extremity”) AND (“rehabilitation” OR “physical therapy”)	Research articles	143 results
Springer Nature Link	(“robotic gait training” OR “robot-assisted”) AND (“body weight support” OR “dynamic body weight support”) AND (“lower limb” OR “lower extremity”) AND (“fracture” OR “trauma”) AND (“rehabilitation” OR “physical therapy”)	Article, English	540 results
Wiley Online Library	(“robotic gait training” OR “robot-assisted”) AND (“body weight support” OR “dynamic body weight support”) AND (“lower limb” OR “lower extremity”) AND (“fracture” OR “trauma”) AND (“rehabilitation” OR “physical therapy”)	English	15 results
SCOPUS	TITLE-ABS-KEY ((“robotic gait training” OR “robot-assisted”) AND (“fracture” OR “trauma”) AND (“rehabilitation” OR “physical therapy”)	Article, English, Rehabilitation	15 results
Total			773 results

**Table 2:** Overview of included studies that have used different types of DBWS systems for gait training in patients with post-traumatic lower limb injuries.

Authors	Country	Year	Study Design	NO. Pac Incl	References
Palke et al. [33]	Germany	2022	Open-label randomized clinical trial	73	[33]
Kim et al. [34]	South Korea	2020	Randomized controlled trial	34	[34]
Ngamwongsanguan et al. [35]	Thailand	2024	Pilot interventional study	10	[35]
Giangregorio et al. [36]	Canada	2009	Pilot non-randomized controlled feasibility study	21	[36]
Chen et al. [37]	China	2024	Randomized controlled trial	52	[37]
Bodine et al. [38]	USA	2025	Case report	1	[38]
Ali et al. [39]	China	2025	Randomized controlled trial	74	[39]

**Table 3:** Score obtained on the PEDro scale [31] of the selected studies.

Study	Eligibility Criteria and Source (1)	Random Allocation (2)	Concealed Allocation (3)	Baseline Similarity (4)	Subject Blinding (5)	Therapist Blinding (6)	Assessor Blinding (7)	Adequate Follow-Up (8)	Intention-to-Treat (9)	Between-Group Statistical Comparisons (10)	Reporting of Point Estimates and Variability (11)	Total Score (2–11)
Palke et al. [33]	yes	yes	no	no	no	no	no	no	yes	yes	yes	4/10
Kim et al. [34]	yes	yes	no	yes	yes	no	yes	no	no	yes	yes	6/10
Ali et al. [39]	yes	no	yes	yes	no	no	yes	yes	no	yes	yes	6/10
Chen et al. [37]	yes	yes	no	yes	no	no	no	no	no	yes	yes	4/10

Scoring: 0–3 are considered ‘poor’, 4–5 ‘fair’, 6–8 ‘good’, and 9–10 ‘excellent’ [31].

**Table 4:** Synthesis of the main characteristics and outcomes of the studies included in the systematic review.

AUTHORS	SAMPLE	AGE (AVERAGE, STANDARD DEV.)	TYPE OF PATHOLOGY	DEVICE–TRAINING PROGRAM	FREQUENCY AND DURATION	OUTCOMES
Palke et al. (2022) [33]	IG = 29 CG = 24	IG: 41 CG: 43	tibial plateau or ankle fractures	antigravity treadmill (Alter G^®^)	first six weeks postoperatively (does not specify the exact frequency or duration of each session on the treadmill)	↑ DGI-IG ↑ FAOS: IG = CG ↑ KOOS: IG = CG ↑ ROM ↓ Muscle Atrophy: IG
Kim et al. (2020) [34]	EG = 17 CG = 17	EG: 48.82 ± 5.96 CG: 51.82 ± 5.91	femoral fracture	EG: An anti-gravity treadmill (version 1.20, model: Anti-gravity Treadmill M320/F320; Altern-G Inc., Fremont, CA, USA) IG: exercise methods of Vanessa and William	20 min/ses 4 weeks	↑ MRC hip extensor: EG ↑ MRC hip flexors: EG = CG ↑ EMG activity: EG
Ngamwongsanguan et al. (2024) [35]	N = 10	75.6 ± 6.2	femoral neck fractures	SensibleSTEP end-effector robotic gait training	30 min/ses 2 times/week 4 weeks	↑ FAC ↓ TUG ↑ FSST ↑ Gait speed (10 m walk) ↑ Stride length ↑ SLS ↑ Cadence ↓ VAS ↓ Gait aid use ↓ Level of assistance
Giangregorio et al. (2009) [36]	IG = 14 CG = 7	IG = 79.9 ± 7.0 CG = 83.7 ± 8.6	hip fractures	body weight-supported treadmill training (BWSTT) with suspension (Pneumex Pneu-weight)	20 min/ses 5 times/week	↑ TUG: IG ↑ 2MWT: IG ↑ LEFS: IG ↓ FES-I: IG ↑ Feasibility ↓ Adverse events
Chen et al. (2024) [37]	OG = 26 CG = 26	OG= 40.73 ± 9.40 CG= 39.73 ± 12.57	pronation–external rotation (PER) type III–IV ankle fractures	OG: standing bed training combined with early anti-gravity running table rehabilitation CG: conventional postoperative rehabilitation	20 min/ses 5 times/week 8 weeks	↑ Bone mineral density: OG ↑ AOFAS ankle–hindfoot score: OG ↓ VAS: OG = CG ↑ Ankle dorsiflexion ROM ↑ Ankle plantarflexion ROM ↓ Adverse events
Bodine et al. (2025) [38]	N = 1	73	multiple fractures of the upper and lower extremities, ribs, pelvis, and sacrum	DBWS system (Vector Gait and Safety System, Bioness) combined with conventional physiotherapy	3 sessions with DBWS	↑ Standing tolerance ↑ Transfer ability ↑ Walking distance ↓ Assistance required for mobility ↓ Adverse events
Ali et al. (2025) [39]	Group A = 34 Group B = 40	GA = 47.36 ± 10.63 GB = 22.63 ± 2.31	pelvic Tile C fractures	GA: robotic-assisted mechanotherapy using the Hocoma Lokomat^®^ combined with conventional physiotherapy GB: conventional rehabilitation	30–60 min/ses 3 times/week 12 weeks	↑ MPS: Group A ↓ VAS: Group A = Group B ↑ Walking ability ↑ Sitting tolerance ↑ MRC hip and knee: Group A ↓ Adverse events

IG: intervention group; CG: control group; EG: experimental group; N: number; OG: observational group; GA: group A; GB: group B; ↑ indicates increase/improvement; ↓ indicates decrease/reduction.

## Data Availability

All data supporting the findings of this study are available in the original articles included in the systematic review, which are cited in the reference list. No new data were generated. Further inquiries can be directed to the corresponding authors.

## Supplementary Materials

The following supporting information can be downloaded at: https://www.mdpi.com/article/10.3390/medicina62030498/s1, PRISMA 2020 Main Checklist.

## Abbreviations

The following abbreviations are used in this manuscript:

DBWS	Dynamic Body Weight Support
PRISMA	Preferred Reporting Items for Systematic Reviews and Meta-Analyses
ROM	Range of Motion
MRC	Medical Research Council
FAC	Functional Ambulation Categories
FIM	Functional Independence Measure
TT	Tinetti Test
ADL	Activities of Daily Living
IADL	Instrumental Activities of Daily Living
PEDro	Physiotherapy Evidence Database
RoB 2	Cochrane Risk of Bias 2
RCTs	Randomized controlled trials
FAOS	Foot and Ankle Outcome Score
KOOS	Knee Injury and Osteoarthritis Outcome Score
DGI	Dynamic Gait Index
ORIF	Open Reduction and Internal Fixation
THR	Total Hip Replacement
TUG	Time Up and Go
SLS	Single Leg Stance
FSST	Four Step Square Test
VAS	Visual Analog Scale
BWSTT	Body Weight Support Treadmill Training
2MWT	2-Minute Walk Test
LEFS	Lower Extremity Functional Scale
FES-I	Falls Self-Efficacy Scale
PER	Pronation External Rotation
AOFAS	American Orthopaedic Foot and Ankle Society
MPS	Majeed Pelvic Score
EMG	Electromyography
QOL	Quality of Life
BWS	Body Weight Support
VR	Virtual Reality
